# Isolation, identification, and metabolic phenotyping of *Alcaligenes faecalis* Zr60, an antagonistic bacterium against tobacco brown spot disease

**DOI:** 10.3389/fmicb.2026.1725042

**Published:** 2026-06-05

**Authors:** Lin Zhang, Ligang Xiang, Xinghong Zhang

**Affiliations:** 1 College of Agriculture, Guizhou University, Guiyang, China; 2 Guizhou Province Tobacco Company Zunyi Branch, Zunyi, Guizhou, China

**Keywords:** *Alcaligenes faecalis*, antagonistic activity, biological properties, growth-promoting activity, metabolic phenotype, tobacco brown spot disease

## Abstract

To identify effective biocontrol bacteria antagonistic to tobacco brown spot disease, we isolated and screened bacterial strains from tobacco rhizospheric soil using dilution plating and plate confrontation methods, with *Alternaria alternata* as the indicator organism. The antimicrobial activity of fermentation broth was determined using the mycelial growth rate method. Strain identification involved morphological observations and 16S rRNA sequencing. The strains were evaluated for broad-spectrum antimicrobial activity, growth-promoting properties, biological characteristics, and metabolic phenotypic features. Among the 113 strains isolated, Zr60 showed strong antagonism against tobacco brown spot disease, with inhibition rates of 81.93 and 87.47% in plate confrontation and fermentation broth tests, respectively. Zr60 was identified as *Alcaligenes faecalis* through morphological analysis and 16S rRNA sequencing. This strain exhibited significant broad-spectrum antagonistic activity against eight phytopathogens, with an average inhibition rate of 75.64%. Growth-promoting properties and biological characteristics indicated that Zr60 solubilized inorganic phosphorus and produced siderophores. Optimal growth temperature was 28 °C, and the optimal culture medium was Luria–Bertani broth. Metabolic phenotype analysis revealed that Zr60 metabolized 95% of carbon, 94% of nitrogen, and 100% of phosphorus and sulfur sources, encompassing 94 biosynthetic pathways. It demonstrated strong environmental adaptability, surviving in high-concentration osmotic solutions. It proliferated within a pH range of 5–10 (optimal pH 9) and exhibited amino acid decarboxylase and deaminase activities. These findings highlight the broad nutritional versatility and exceptional environmental adaptability of *A. faecalis* Zr60. This study provides a theoretical basis for the potential application of this strain in controlling tobacco brown spot disease.

## Introduction

Tobacco brown spot disease, caused by *Alternaria alternata*, is a highly destructive fungal disease that primarily affects mature tobacco leaves. It can severely impair the growth of flue-cured tobacco, reducing yield and quality and resulting in significant economic losses (Chen et al., 2001). The primary control strategies include chemical, agricultural, and biological methods. Although chemical control is widely used and effective, concerns about pesticide residues and pathogen resistance are increasing (Yang et al., 2021). Therefore, low-cost, residue-free, non-toxic, and efficient biological control methods have become a major focus in managing tobacco brown spot disease (Li et al., 2020). Currently, commonly employed biocontrol agents include *Trichoderma* spp., *Bacillus* spp., and *Streptomyces* spp. (Zhao et al., 2022). *Alcaligenes faecalis* (family Alcaligenaceae) is a gram-negative bacterium that is widely distributed in soil and aquatic environments (Nakano et al., 2013).

The Alcaligenaceae family belonging to the class Betaproteobacteria, encompasses several genera, such as *Alcaligenes*, *Achromobacter*, and *Bordetella*, whose members are typically rod-shaped (Willems, 2014). Alcaligenaceae species can utilize various organic compounds—including carbohydrates, amino acids, and organic acids—demonstrating strong metabolic capabilities. Additionally, they possess powerful degradative abilities for compounds such as aromatic hydrocarbons (Nojiri et al., 1999). These bacteria also generate secondary metabolites with antifungal and antibacterial activities, which further suppress the growth of pathogenic microorganisms. Moreover, they can promote plant growth by synthesizing plant hormones, such as indole-3-acetic acid (IAA), which enhances root development and nutrient uptake (Glick, 2012). *A. faecalis* is effective in treating pig farm wastewater with high ammonium levels under aerobic conditions (Joo et al., 2006). Shoda and Ishikawa (2014) leveraged the nitrification and denitrification capabilities of this bacterium to treat elevated ammonium concentrations in sludge from anaerobic nitrification reactors. It can also degrade various compounds, including monocyclic and polycyclic aromatic hydrocarbons, toxic pesticides, sulfonamide drugs, and certain inorganic compounds (Zhang, 2017). In particular, it degrades phenol, cyanide, quinoline, and picolinic acid. Wang et al. (2016) reported that *A. faecalis* LY2 could degrade phenol at concentrations of 50–500 ppm, while Kong et al. (2014) found that *A. faecalis* JBW4 degraded the potent insecticide endosulfan in soil through a non-oxidative pathway, thereby reducing the genotoxicity and ecotoxicity of contaminated soils. *A. faecalis* shows broad application potential in bioremediation and industrial production. It can synthesize non-natural amino acids, which are widely used in pharmaceutical industry, and exhibits nematicidal and antibacterial activities. Plant-parasitic nematodes are major agricultural pathogens that reduce crop yields and cause significant economic losses. Zhou (2005) isolated a nematode-antagonistic strain of *A. faecalis* from rhizospheric soil, providing a theoretical basis for developing microbial nematicides. Hernández-Mendoza et al. (2014) reported that an *A. faecalis* strain could rapidly colonize insect-pathogenic nematodes and kill insects. Sayyed and Chincholkar (2009) investigated antifungal activity in siderophore-producing *A. faecalis*, which inhibits pathogens by competing for iron ions, offering a sustainable approach to integrated plant disease management. Yokoyama et al. (2013) isolated *A. faecalis* AD15, which suppressed plant pathogens by producing hydroxylamine, highlighting its potential as a biopesticide. Bharali et al. (2011) demonstrated that a thermophilic *A. faecalis* strain produced biosurfactants using diesel oil as the sole carbon (C) source; the partially purified surfactant exhibited antimicrobial activity. Kapley et al. (2016) discovered that *Alcaligenes* spp. HPC1271 produced the antibiotic echinomycin. Liu et al. (2016) also reported that *A. faecalis* NBIB-017 harbors numerous genes encoding antibacterial factors. Xu et al. (2015) isolated seven compounds from *A. faecalis* YMF 3.175 and confirmed their antibacterial activities.

Biolog phenotype microarray (PM) technology, developed by Bochner in 2000, is a tool for measuring cell phenotypes and is considered complementary to genomics, proteomics, and metabolomics (Bochner et al., 2001; Bochner, 2003). By analyzing the metabolic fingerprints of microorganisms on a chip, microbes can be screened for adaptability and metabolic capabilities, such as antibiotic production or high nutritional competitiveness. Each well of a 96-well microplate contains a dried culture substrate and a chromogen. When microbial suspensions are added and incubated, phenotypic traits manifest as color changes, producing kinetic curves that quantitatively measure reaction intensity. Various PM plates are coated with substrates essential for growth, enabling tests under different conditions, including assessments of carbon (C), nitrogen (N), phosphorus (P), and sulfur (S) utilization. This technology allows the evaluation of microbial metabolic activity under diverse nutrient sources, pH levels, and osmotic pressures, thereby optimizing culture conditions. Garcia and Chen (2020) used the Biolog PM system to characterize the metabolic traits of biocontrol bacteria. However, only limited studies have examined the antagonistic effects of *A. faecalis*, comprehensively analyzed its C, N, S, and P requirements, and evaluated its adaptability to diverse environmental conditions.

Herein, rhizosphere soil was collected from tobacco fields in Fuquan, Guizhou Province. A bacterial strain, designated Zr60, with antagonistic activity against the tobacco brown spot pathogen, was screened and isolated. The strain was identified using a combination of morphological observations and molecular methods. Its antimicrobial spectrum, growth-promoting activity, and biological characteristics were evaluated. Additionally, its metabolic phenotype was analyzed using PM technology. This study provides a theoretical foundation for the discovery and application of biocontrol strains against tobacco brown spot disease.

## Methods

### Test strains

An antagonistic strain was isolated from tobacco rhizospheric soil in the Fuquan tobacco-growing region of Guizhou Province, China (26°44′N, 107°21′E). The strain was designated Zr60 and is preserved in the Microbiology Laboratory 1,201 of the Guizhou Academy of Tobacco Science. The target pathogen strains—*Fusarium graminearum*, *Pectobacterium carotovorum*, *Ceratobasidium cornigerum*, *Colletotrichum gloeosporioides*, *Thielaviopsis basicola*, *Fusarium pseudograminearum*, *Rhizoctonia solani*, *Bipolaris zeicola*, and *Alternaria alternata*—are also preserved in the same laboratory.

### Media and reagents

The following media were used: nutrient agar (NA), potato dextrose agar (PDA), Luria–Bertani (LB) Broth, tryptic soy broth (TSB), beef extract peptone broth (BEPB), nutrient broth (NB), potato dextrose broth (PDB), YE broth (YEB), Ashby’s medium, organophosphorus agar medium, inorganic P agar medium, potassium bacteria agar (PBA) medium, and CAS agar medium. The antagonistic strain was identified using BUG + B medium, which consists of Biolog Universal Growth (BUG) medium (Biolog, USA) supplemented with lyophilized defibrillated sheep blood (B) (Beijing Youkangjiye Biotechnology Co., China). Metabolic plates PM1–10, inocula IF-0a GN/GP (#72268), IF-10b GN/GP (#72266), and dye MixA (#74221) were obtained from Biolog (USA). The Bacterial Genomic DNA Extraction Kit (Product ID: 12224–50) was obtained from QIAGEN (Germany).

### Isolation of the antagonistic strains

Soil samples from the tobacco rhizosphere were processed using serial dilution and spread plate methods (Oku et al., 2011). Briefly, 10 g of soil was placed in a 250-mL Erlenmeyer flask containing 90 mL of sterile water and shaken at 150 rpm for 20 min on a rotary shaker. After brief settling, 1 mL of the soil suspension was transferred to a test tube containing 9 mL of sterile water and mixed thoroughly to obtain a 10^−2^ dilution. This process was repeated to prepare 10^−3^, 10^−4^, and 10^−5^ dilutions. A 0.2-mL aliquot from each dilution was spread on NA plates, which were incubated in an inverted position at 37 °C for 48 h. Colonies with distinct morphologies and sizes were selected, and purified strains were inoculated onto slant media for preservation.

### Screening of bacteria antagonistic to tobacco brown spot disease

Initial screening of the isolated bacteria for antagonistic activity was performed using the plate confrontation method (Xiao et al., 2022). *A. alternata* was cultured on PDA plates for 3 days. Sterile 5-mm-diameter plugs were taken from the colony margins and inoculated at the center of fresh PDA plates. The isolated and activated bacterial strains were then spot-inoculated at three equidistant points, each 2 cm from the *A. alternata* disc. Sterile water served as the control. After incubation at 28 °C for 5 days, the diameter of the *A. alternata* colonies was measured using the cross-measurement method. Antifungal activity of the bacteria was expressed as the inhibition rate, calculated as follows:


$$\begin{array}{l} \text{Inhibition rate}\;(\%)=\\ (\frac{\text{Control Colony Diameter}-\text{Treated Colony Diameter}}{\text{Control Colony Diameter}})\times 100\% \end{array}$$

### Bacteriostatic activity of the Zr60 fermentation broth

Zr60, which exhibited the strongest inhibitory effect against *A. alternata*, was selected for further experiments. The mycelial growth rate method was applied (Yu et al., 2022). Zr60 was cultured in LB broth at 28 °C with shaking at 180 rpm for 72 h to obtain the fermentation broth. The broth was then centrifuged at 8,000 rpm for 10 min at 4 °C. The supernatant was collected and filtered through a 0.22-μm bacterial filter to obtain the sterile fermentation filtrate. This filtrate was thoroughly mixed with PDA at a 1:9 volume ratio, cooled to approximately 55 °C, and poured into Petri dishes. An *A. alternata* disc was then placed at the center of each PDA plate. Each treatment was performed in triplicate, with plates lacking the sterile fermentation filtrate serving as controls. The plates were incubated at 28 °C for 5 days. The inhibition rate of the sterile Zr60 fermentation filtrate against *A. alternata* was calculated as follows:


$$\begin{array}{l} \text{Inhibition rate}\;(\%)=\\ (\frac{\text{Control Colony Diameter}-\text{Treated Colony Diameter}}{\text{Control Colony Diameter}})\times 100\% \end{array}$$

### Identification of antagonistic bacteria

#### Observation of Zr60 colonies

The colony morphology of Zr60 was observed following the method of Kauffmann et al. (2004). Zr60 was activated on NA plates and incubated at 28 °C for 24 h. Colony characteristics, including size, shape, color, and transparency, were recorded.

#### Molecular identification and phylogenetic analysis of Zr60

Genomic DNA of Zr60 was extracted for PCR amplification using a commercial DNA extraction kit, following the manufacturer’s instructions. Molecular identification of the antagonistic bacterium was performed via 16S rRNA sequence homology analysis. Using the method described by Osborne et al. (2010), universal bacterial primers 27F and 1492R were employed for amplification. The resulting amplicons were sent to Wuhan Boyuan Biotechnology Co., Ltd. for sequencing. The sequences obtained were analyzed using the BLAST program in the GenBank database.^1^ A phylogenetic tree was constructed using the Neighbor-Joining method in MEGA-X software,^2^ based on 16S rRNA gene sequences.

#### Determination of the antimicrobial spectrum of Zr60

The antimicrobial spectrum of Zr60 was determined using test pathogens and the plate confrontation method (Xiao et al., 2022). Pathogens, including *F. graminearum*, *P. carotovorum*, *C. cornigerum*, *C. gloeosporioides*, *T. basicola*, *F. pseudograminearum*, *R. solani*, and *B. zeicola*, were activated and cultured for use. Sterile 5-mm plugs taken from the margins of pathogen cultures were placed at the center of PDA plates. Zr60 was then inoculated at three equidistant points, each 2 cm from the plug, using disposable inoculation loops, and incubated at 28 °C for 5 days. PDA plates inoculated only with pathogen plugs served as controls. Each treatment was performed in triplicate. The diameters of pathogen colonies were measured using the cross-measurement method, and the inhibition rate was calculated as follows:


$$\begin{array}{l} \text{Inhibition rate}\;(\%)=\\ (\frac{\text{Control Colony Diameter}-\text{Treated Colony Diameter}}{\text{Control Colony Diameter}})\times 100\% \end{array}$$

### Growth-promoting activity of Zr60

#### IAA secretion ability of Zr60

The ability of Zr60 to secrete IAA was qualitatively assessed using the Salkowski colorimetric method (Glickmann and Dessaux, 1995). The Salkowski reagent, consisting of concentrated H_2_SO_4_ and FeCl_3_, forms a pink-to-red complex in the presence of IAA. The color intensity is directly proportional to the IAA concentration, allowing qualitative estimation based on color depth. A red coloration of the culture fluid indicated IAA production by the bacterium.

#### N-fixing ability of Zr60

Following the method described by Villar et al. (2017), Zr60 was cultured for 24 h to induce growth. A single colony was then picked with an inoculation loop and streaked onto Ashby’s N-free solid medium plates and incubated at 28 °C for 72 h. Growth on the plates indicated that Zr60 possesses N-fixing ability.

#### P-solubilizing ability of Zr60

Following the method described by Lei et al. (2025), Zr60 was cultured for 24 h to activate growth. A single colony was picked with a sterile inoculation loop and evenly spot-inoculated at five points on inorganic or organic phosphate-solubilizing solid media plates. The plates were incubated at 28 °C for 72 h and observed for the formation of transparent halos around the colonies. The diameters of these halos were measured to qualitatively assess the P-solubilizing ability of Zr60.

#### K-solubilizing ability of Zr60

Per the method described by Yu (2010), Zr60 was cultured for 24 h to activate growth. A single colony was picked with a sterile inoculation loop and evenly spot-inoculated at five points on K-solubilizing solid medium plates. After incubation at 28 °C for 72 h, the formation of transparent halos around the colonies was observed. The diameters of these halos were measured to qualitatively assess the K-solubilizing ability of Zr60.

#### Siderophore production capacity of Zr60

Following the method described by Lei et al. (2020), Zr60 was cultured for 24 h to activate growth. A single colony was picked with a sterile inoculation loop and evenly spot-inoculated at five points on siderophore detection medium plates. After incubation at 28 °C for 72 h, the formation of orange–yellow halos around the colonies was observed. The diameters of these halos were measured to qualitatively assess the siderophore production capacity of Zr60.

### Biological properties of Zr60

#### Inoculum preparation

Zr60 was activated on NA plates and incubated at 28 °C for 24 h. A single colony from the activated culture was picked with a sterile inoculation loop and transferred into 100 mL of LB broth. The culture was incubated at 28 °C with shaking at 180 rpm for 24 h. OD_600_ was measured with a spectrophotometer, and the bacterial suspension was used as the inoculum when the OD_600_ value reached 1.0.

#### Growth curve of Zr60

Following the method described by Wang et al. (2014), the prepared seed culture of Zr60 was inoculated into 100 mL of LB broth at a 1% inoculation ratio. The culture was incubated at 28 °C with shaking at 180 rpm, and OD_600_ was measured every 6 h for 48 h. The experiment was performed in triplicate, with uninoculated medium serving as the control. A growth curve was constructed by plotting incubation time on the x-axis and OD_600_ values on the y-axis to establish the relationship between OD_600_ and viable cell count.

#### Effect of different media on Zr60 growth

Following the method described by Song et al. (2023), the prepared Zr60 suspension was inoculated into 100 mL of TSB, NB, PDB, LB, YEB, or BEPB in Erlenmeyer flasks at a 1% inoculation ratio. Each treatment was performed in triplicate, with uninoculated LB broth serving as the control. The cultures were incubated on a shaker at 28 °C and 180 rpm for 24 h, after which the OD_600_ of the bacterial cultures was measured using a spectrophotometer.

#### Effect of different temperatures on Zr60 growth

Following the method described by Teng et al. (2024), the prepared suspension of Zr60 was inoculated into 100 mL of LB medium in Erlenmeyer flasks at a 1% inoculation ratio. The cultures were incubated on a shaker at 180 rpm at temperatures of 23, 28, 33, 38, 43, and 48 °C for 24 h. Each treatment was performed in triplicate, with uninoculated LB broth serving as the control. The OD_600_ of the bacterial cultures was measured using a spectrophotometer.

### Phenomics analysis of Zr60

Metabolic phenotyping of the antagonistic bacterium Zr60 was performed according to the standard procedure for Biolog gram-negative bacteria (Bochner, 2003). The phenotyping plates were prepared by first activating Zr60 on BUG + B plates, followed by preparation of a PM1–10 inoculum and a suspension of fresh Zr60 cells. The suspension concentration was adjusted to 85% T (T = standard Biolog concentration unit). Plates were incubated in an OmniLog thermostatic incubator at 33 °C for 48 h. OmniLog software was configured, and the metabolic phenotyping data of Zr60 were collected using the Biolog D5E_OKA_data.exe software. The metabolic profiles were analyzed by examining the kinetic curves generated from Zr60 metabolic activity. To assess metabolic activity with C sources, a 22-mL aliquot of the Zr60 suspension was pipetted into a sterile spiking tank and transferred into PM1–2 plates using an 8-channel electronic pipette (100 μL per well). To assess the metabolic activity of Zr60 in N, S, and P sources and identify the biosynthetic pathways involved, a mixed solution of 680 μL containing 2 mol/L sodium succinate and 200 μmol/L ferric citrate was prepared, combined with 68 mL of Zr60 suspension, and transferred into PM3–8 plates (100 μL per well). For assessing osmotic pressure and pH, 100 μL of Zr60 suspension was mixed with 20 mL of IF-10b + dye and transferred into PM9–10 plates (100 μL per well), using an 8-channel electronic pipette.

### Data analysis

IBM SPSS Statistics 23 (IBM Corp., Armonk, NY, USA) was used for data analysis. Differences were considered statistically significant at *p* ≤ 0.05.

## Results

### Isolation of antagonistic strains and determination of antagonistic activity

**Figure 1:**
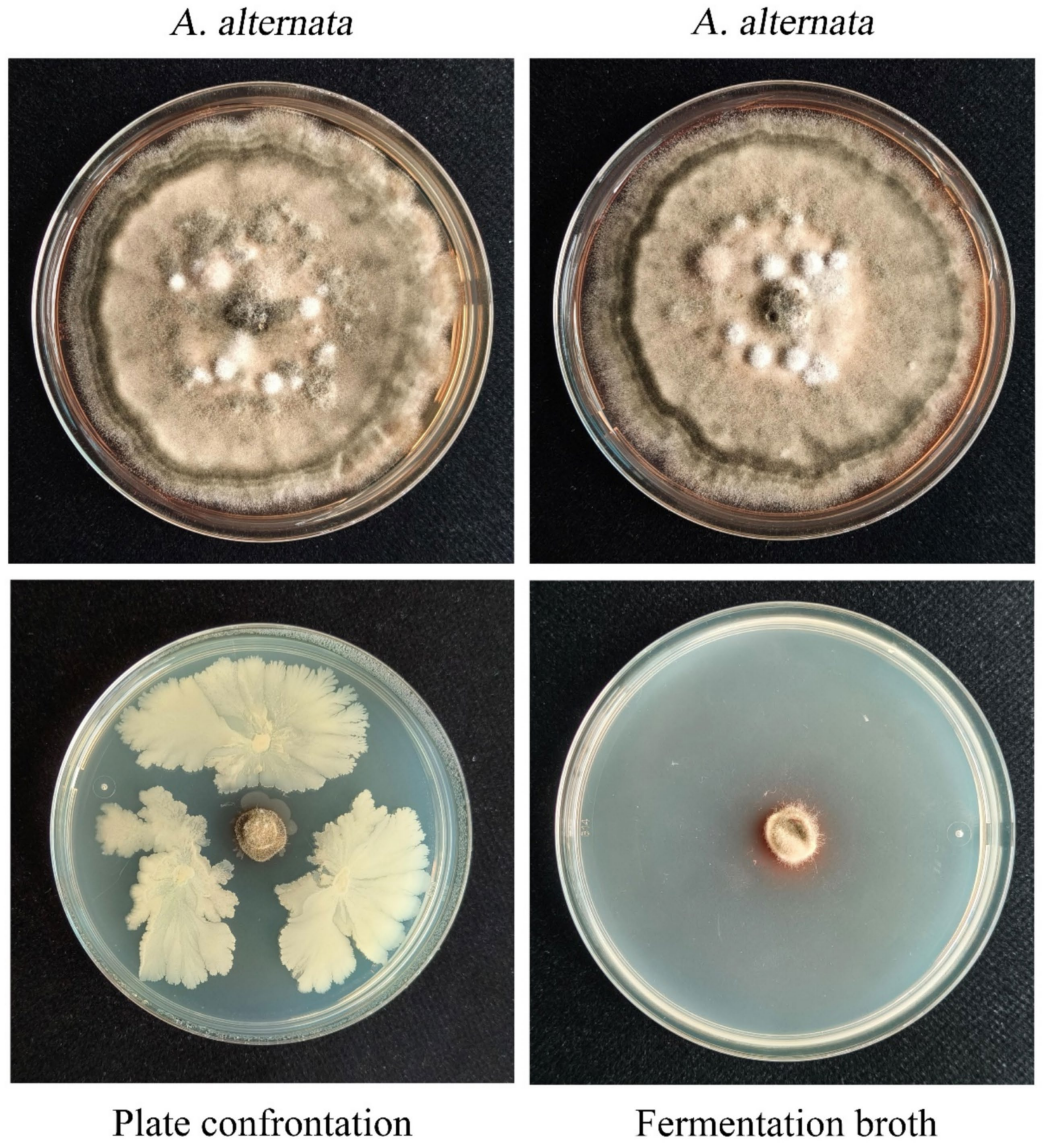
Antagonistic activity of strain Zr60 against *Alternaria alternata* determined using plate confrontation and fermentation broth assays.

**Figure 2:**
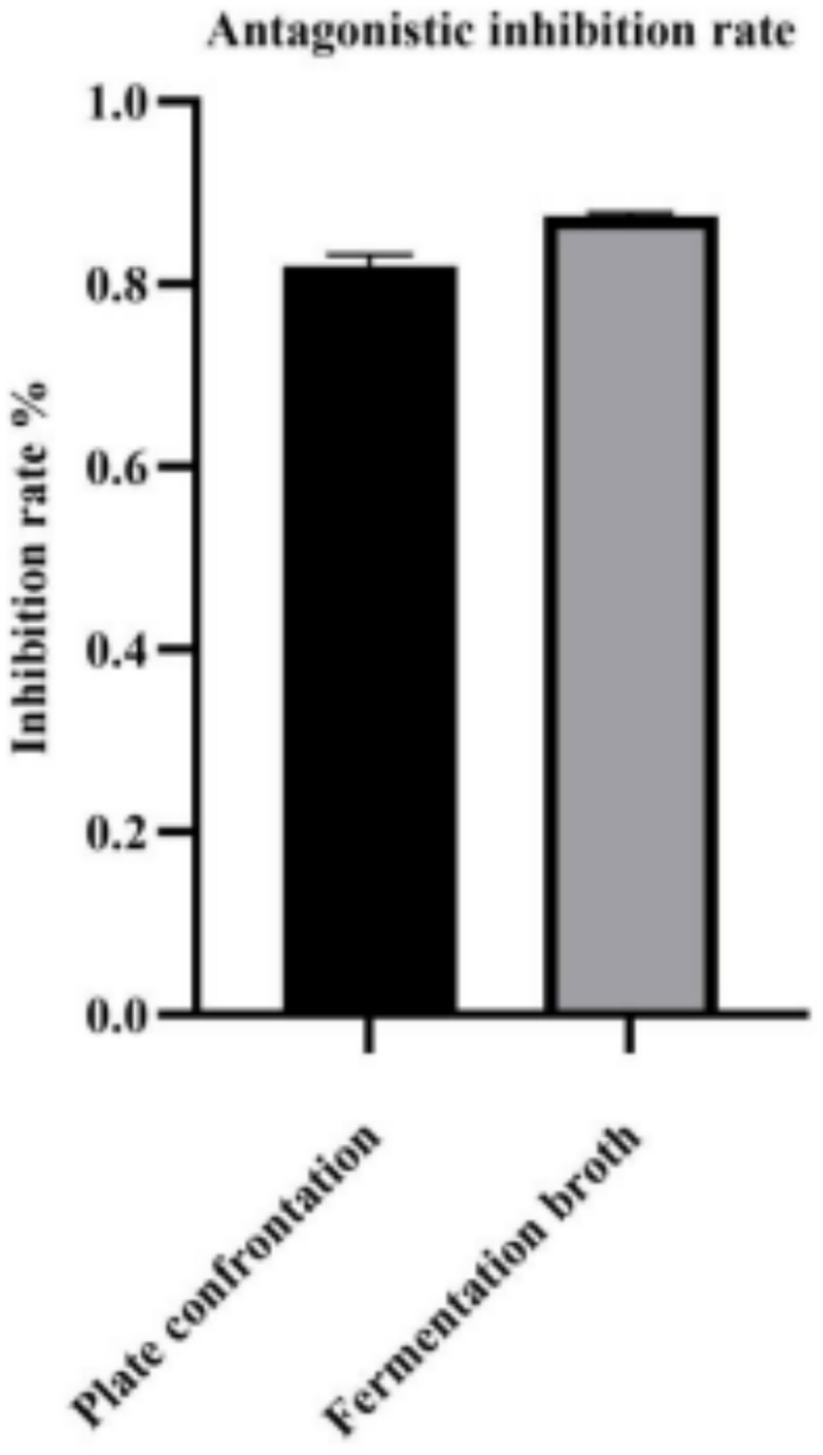
Antimicrobial activity of strain Zr60 against *A. alternata* determined using plate confrontation and fermentation broth assays.

In total, 113 bacterial strains were isolated from the rhizospheric soil of tobacco plants in the Guizhou tobacco cultivation region. *A. alternata* was used as the indicator pathogen to screen for bacterial isolates exhibiting antagonistic activity against tobacco brown spot disease. Among all isolates, strain Zr60 showed the strongest inhibitory effect, with inhibition rates of 81.93% in plate confrontation and 87.47% in fermentation broth assays (Figures 1, 2; Table 1).

**Table 1:** Antimicrobial effect of Zr60 against *Alternaria alternata.*

Treatment	Average inhibition rate (%)	Pathogen diameter (cm)
Plate confrontation	81.93 ± 0.01^b^	1.47 ± 0.06^a^
Fermentation broth	87.47 ± 0.01^a^	1.02 ± 0.02^b^

Different lowercase letters in the table indicate significant differences (*p* ≤ 0.05).

### Identification of Zr60

#### Colony morphology of Zr60

**Figure 3:**
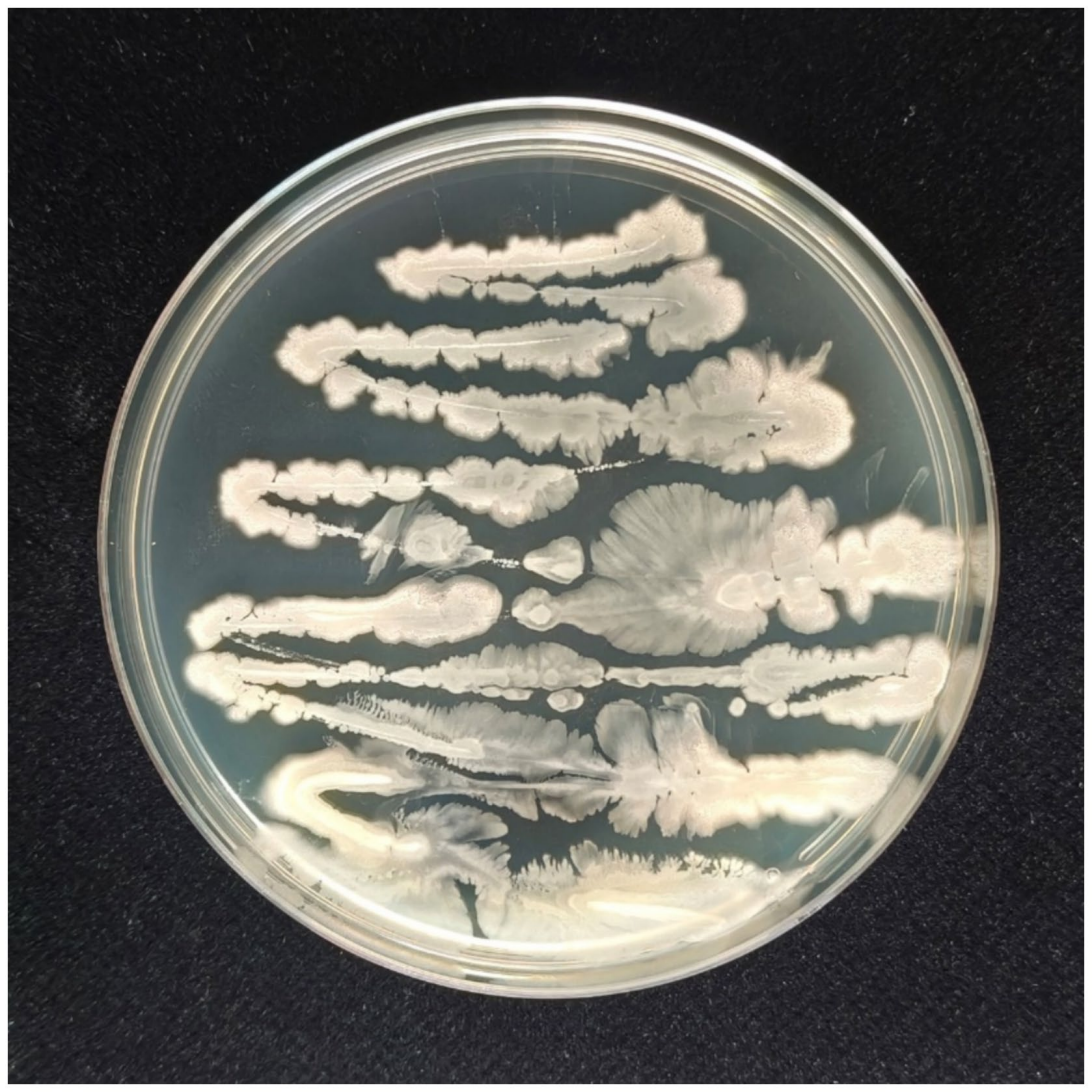
Morphological characteristics of strain Zr60.

Colonies of Zr60 grown on NA medium were light yellow, translucent, round, and 1–2 mm in diameter. They exhibited a slightly raised profile, smooth surface, moist and sticky texture, and neat edges (Figure 3). Gram staining revealed Zr60 to be gram-negative. Microscopic examination showed single, rod-shaped, non-spore-forming cells, consistent with characteristics of the genus *Alcaligenes*.

#### Molecular identification and phylogenetic analysis of Zr60

**Figure 4:**
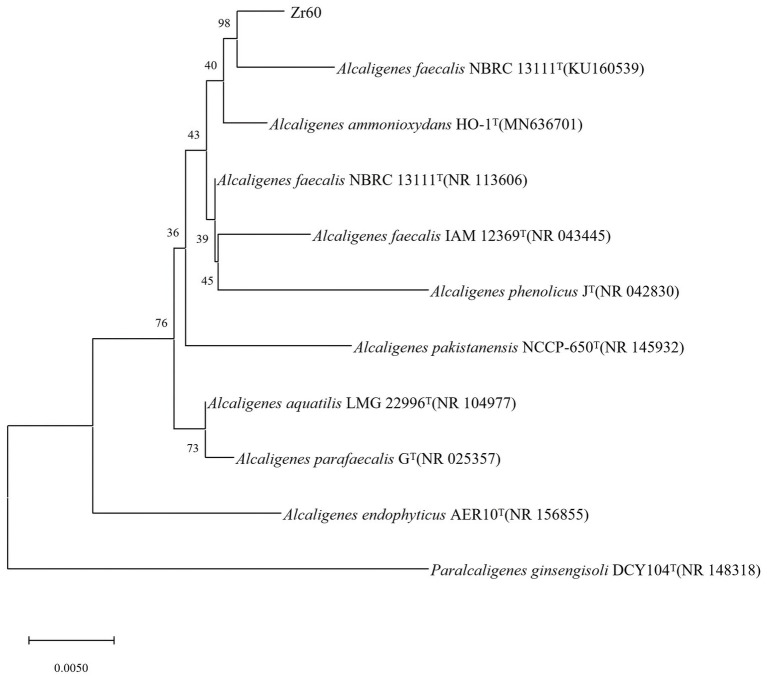
Phylogenetic analysis of strain Zr60 compared with other members of the genus *Alcaligenes*.

The 16S rRNA sequence of Zr60 was determined to be 1,394 bp (GenBank accession number: PP535405.1). Phylogenetic analysis based on 16S rRNA sequences showed that Zr60 clustered with *A. faecalis*, with 98% sequence similarity (Figure 4). Considering this high sequence similarity and morphological characteristics, strain Zr60 was identified as *A. faecalis*.

#### Antimicrobial spectrum of Zr60

**Figure 5:**
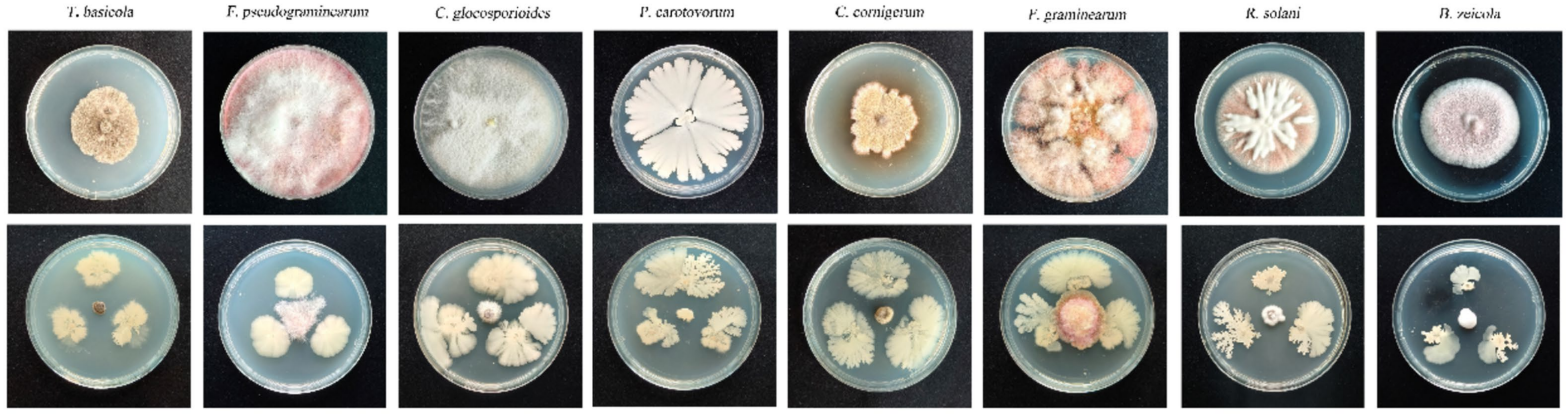
Antimicrobial spectrum of *A. faecalis* Zr60 against various plant pathogens.

**Figure 6:**
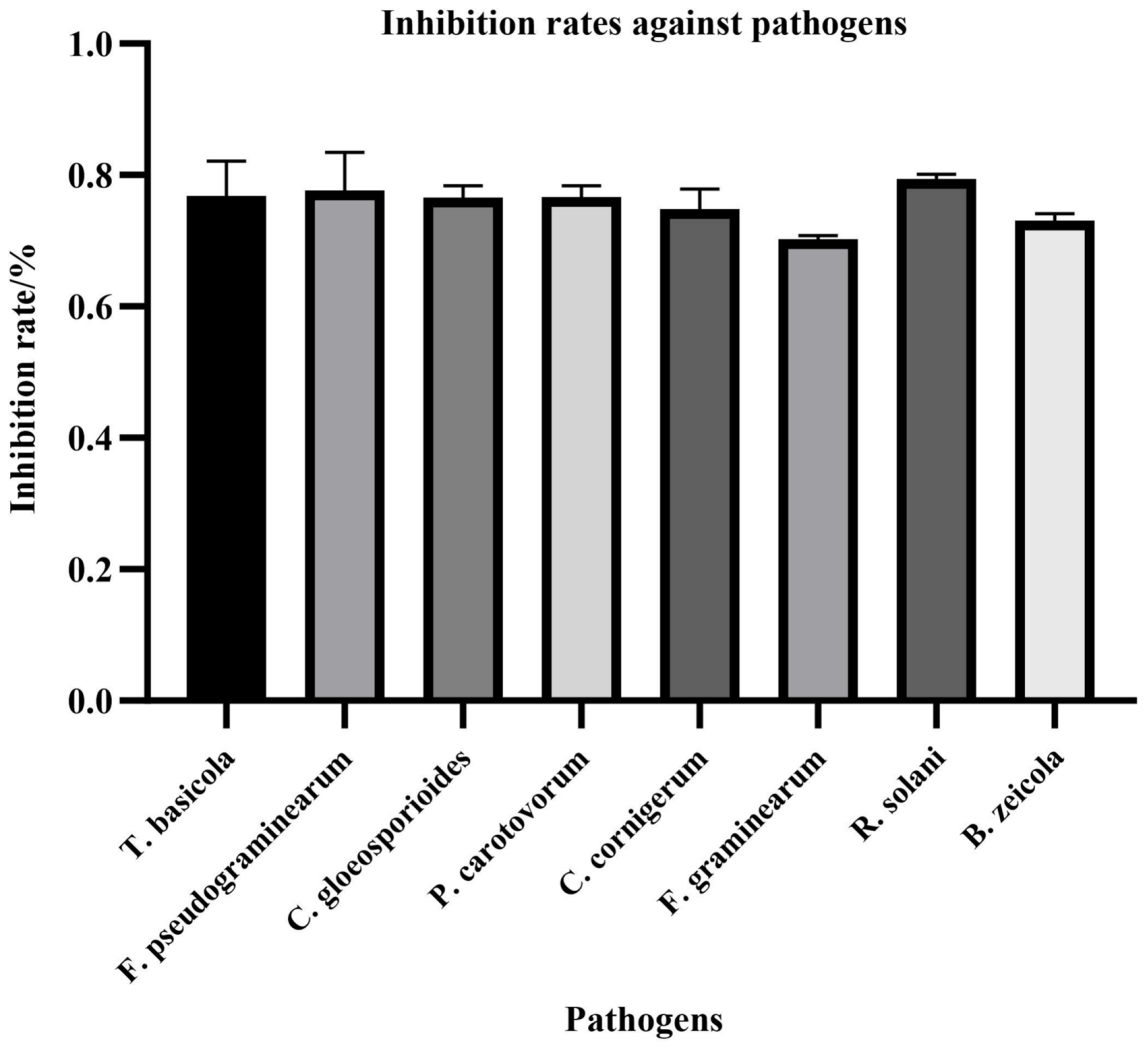
Broad-spectrum antimicrobial activity of strain Zr60.

Bacterial inhibition assays demonstrated that Zr60 suppressed multiple phytopathogens, including *T. basicola*, *F. pseudograminearum*, *C. gloeosporioides*, *P. carotovorum*, *C. cornigerum*, *F. graminearum*, *R. solani*, and *B. zeicola* (Figures 5, 6; Table 2). The corresponding mycelial growth inhibition rates were 76.83, 77.65, 76.54, 76.62, 74.80, 70.24, 79.37, and 73.03%, respectively.

**Table 2:** Inhibition rates of Zr60 against various pathogens.

Pathogen	Inhibition rate (%)
*T. basicola*	76.83 ± 0.03^ab^
*F. pseudograminearum*	77.65 ± 0.03^ab^
*C. gloeosporioides*	76.54 ± 0.01^ab^
*P. carotovorum*	76.62 ± 0.01^ab^
*C. cornigerum*	74.80 ± 0.02^abc^
*F. graminearum*	70.24 ± 0.01^c^
*R. solani*	79.37 ± 0.01^a^
*B. zeicola*	73.03 ± 0.01^bc^

Different lowercase letters in the table indicate significant differences (*p* ≤ 0.05).

#### Functional characterization of Zr60

**Figure 7:**
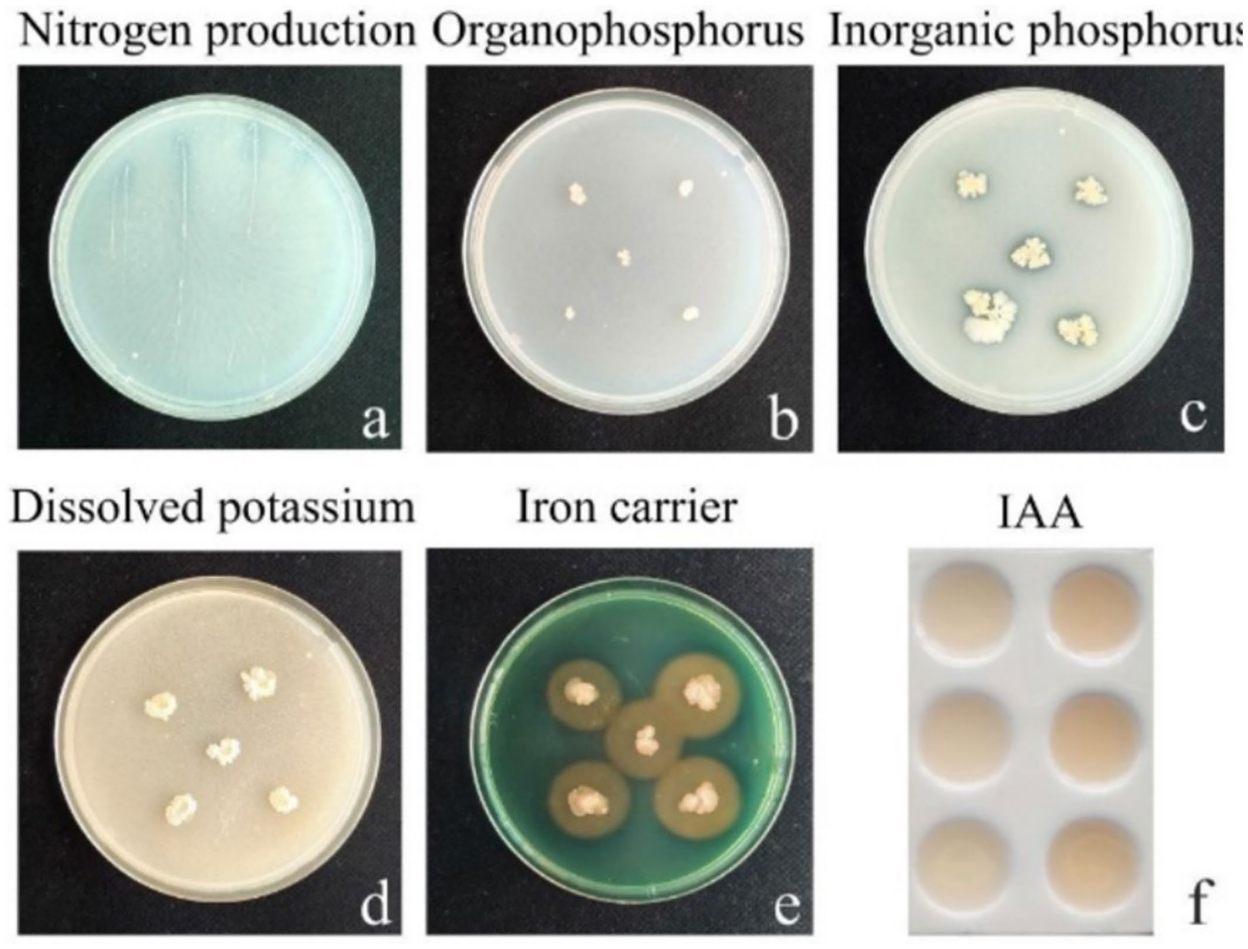
Growth-promoting activities of *A. faecalis* Zr60. **(a)** Nitrogen-fixing capacity. **(b)** Organic phosphorus-solubilizing capacity. **(c)** Inorganic phosphorus-solubilizing capacity. **(d)** Potassium-solubilizing capacity. **(e)** Siderophore production capacity. **(f)** Indole-3-acetic acid production capacity.

On solid inorganic P medium, Zr60 formed hyaline halos with an average diameter of 1.15 cm, indicating its ability to solubilize inorganic P (Figure 7c). Similarly, orange–yellow halos were observed on CAS agar, with a mean diameter of 2.5 cm (Figure 7e), suggesting siderophore production. However, Zr60 did not grow on Ashby’s medium, indicating an inability to fix N (Figure 7a). No transparent halos were observed on organophosphorus agar or PBA, suggesting an inability to solubilize organic P or potassium (Figures 7b,d). On the IAA detection plate, the control liquid remained unchanged, and the treatment liquid did not turn red, confirming the inability to produce IAA (Figure 7f).

#### Biological properties of Zr60

**Figure 8:**
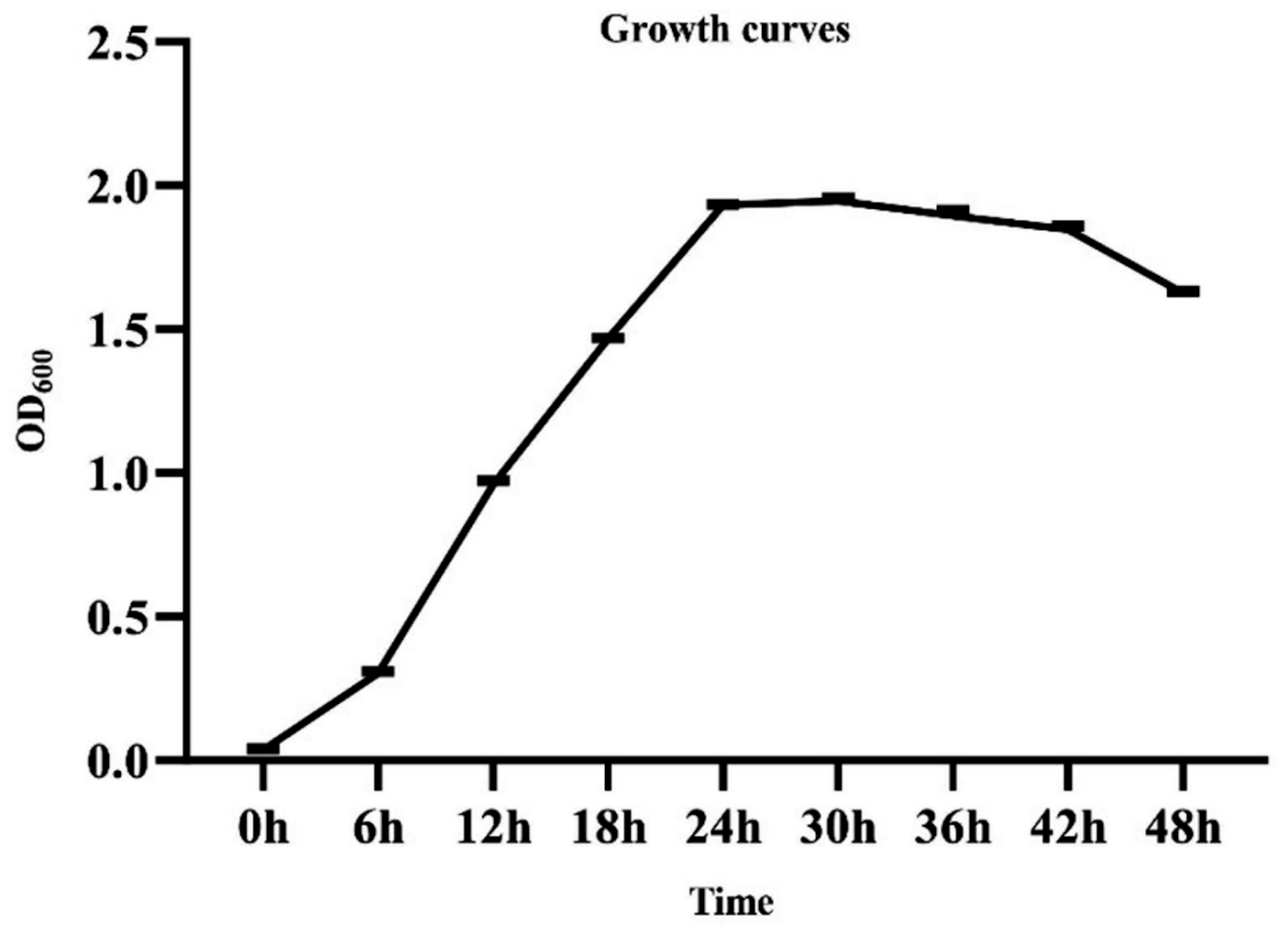
Growth curve of strain Zr60 showing different growth phases over time.

**Figure 9:**
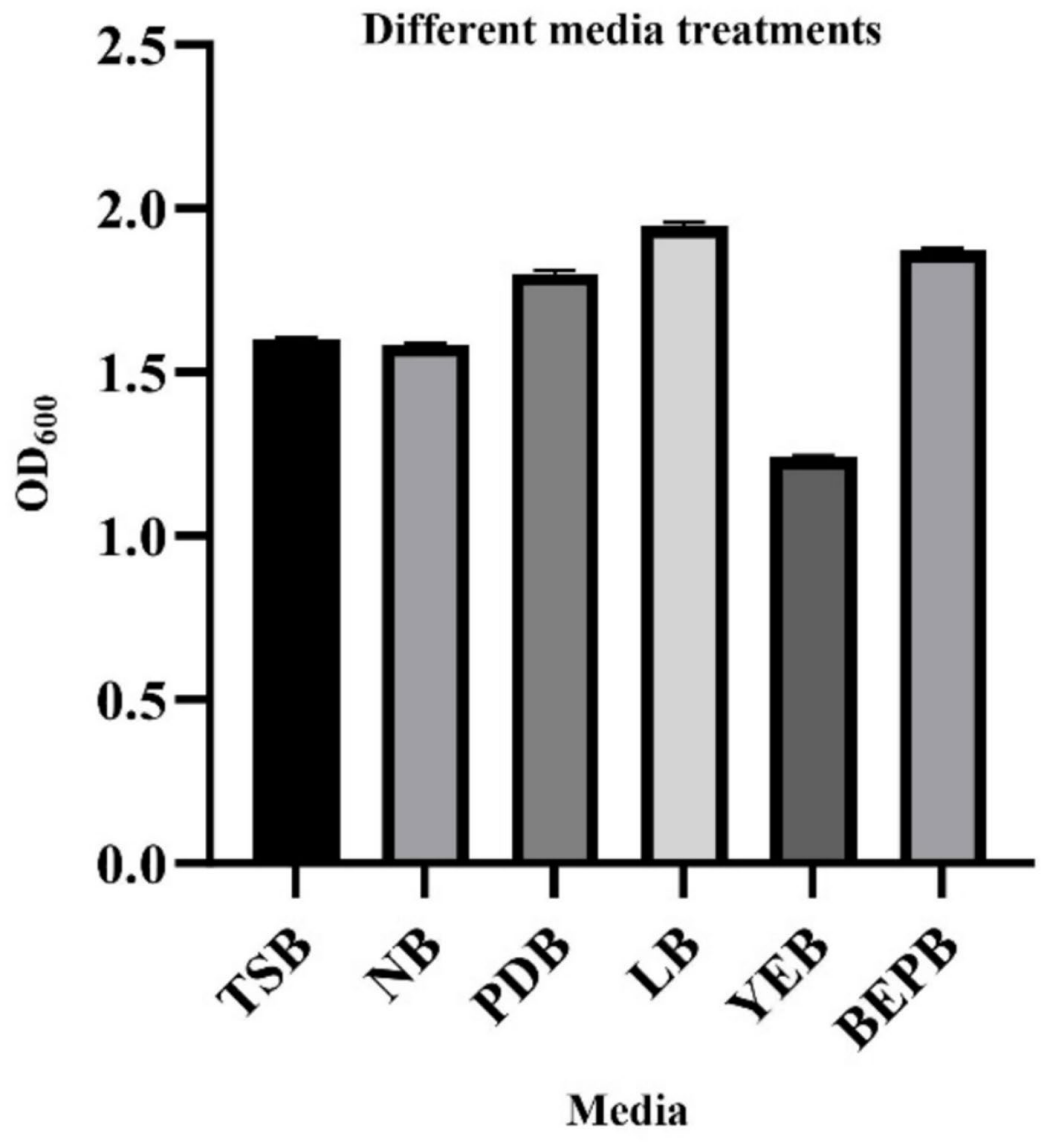
Growth of strain Zr60 in different media.

**Figure 10:**
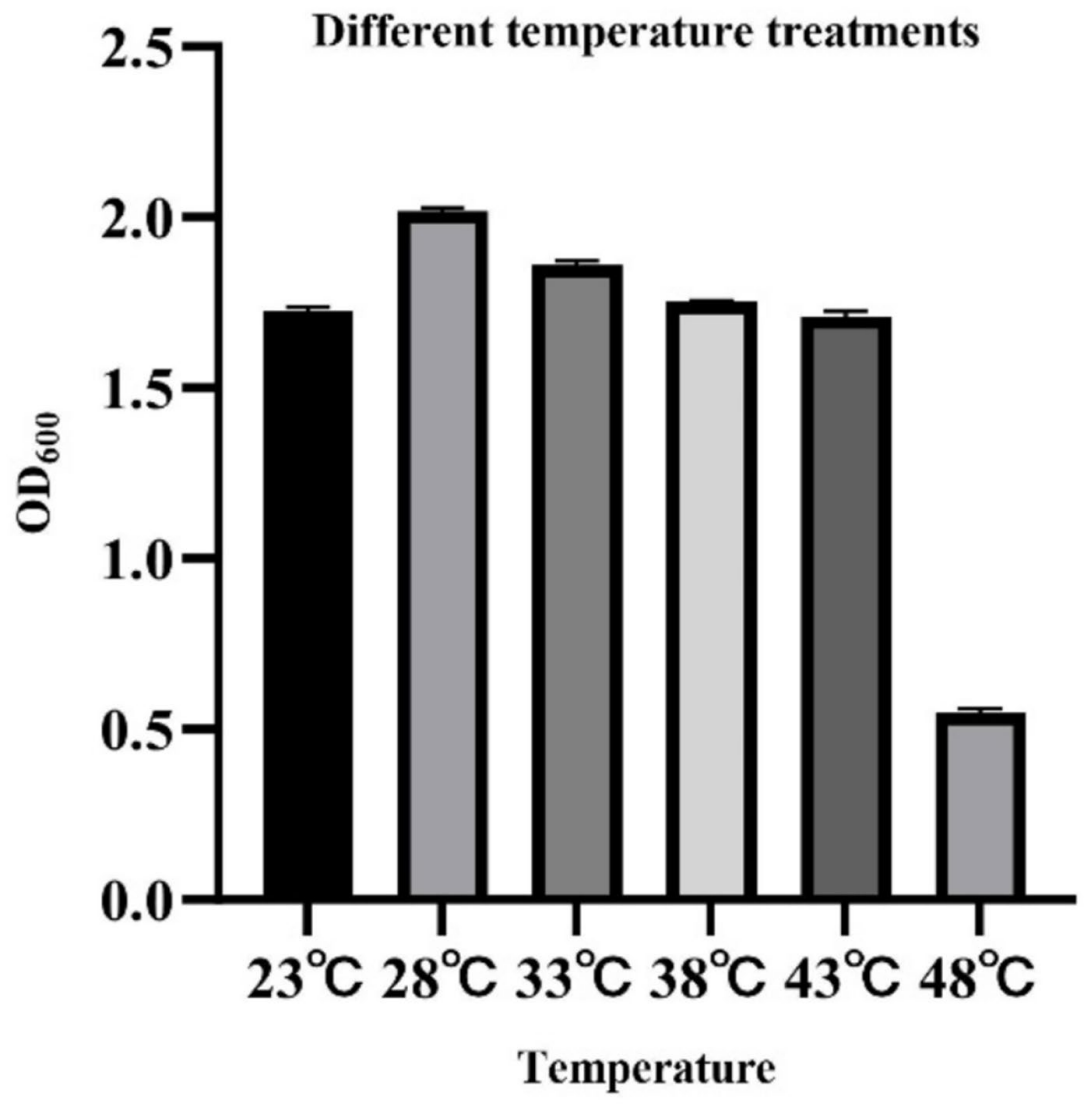
Growth of strain Zr60 at different temperatures.

Figure 8 shows that Zr60 entered the logarithmic phase after a 4-h lag period, reaching peak growth at 24 h, after which growth gradually declined. Zr60 grew well on all tested media, with LB medium supporting optimal growth (Figure 9). It could grow across a temperature range of 23 °C–48 °C, with 28 °C identified as optimal (Figure 10).

### Phenomics analysis of Zr60

#### Phenotypic analysis of C source metabolism in Zr60

**Figure 11:**
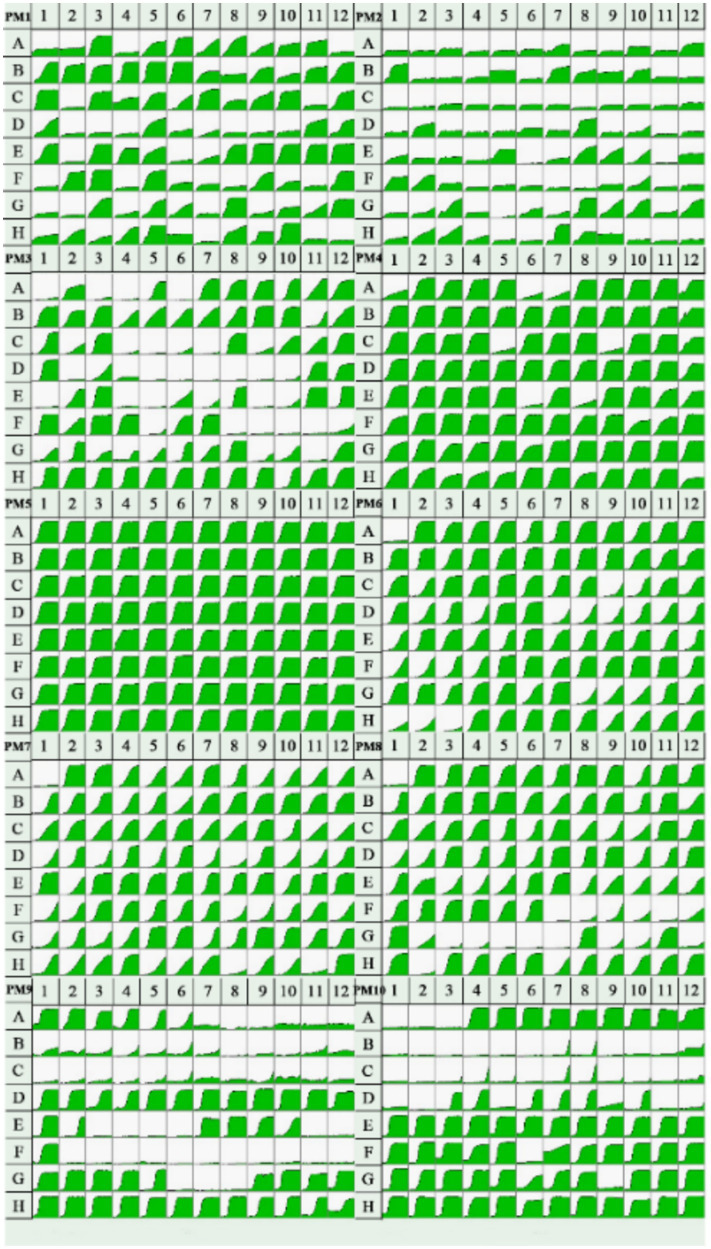
Metabolic profiling of the nutritional requirements and environmental adaptability of *A. faecalis* Zr60.

On the PM1 and PM2 C source assay plates, Zr60 metabolized 181\u00B0C sources (93 on PM1 and 88 on PM2). Among these, 74 were efficiently metabolized, including N-acetyl-D-glucosamine, succinic acid, D-galactose, and L-aspartic acid. In contrast, nine C sources were not metabolized (Figure 11, PM1–PM2, Table 3).

**Table 3:** Substrates in PM1 and PM2 microplates effectively metabolized by *A. faecalis* Zr60.

Well	Substrate	Well	Substrate	Well	Substrate
1-A3	N-acetyl-D-glucosamine	1-D1	L-asparagine	1-H4	Tyramine
1-A5	Succinic acid	1-D5	Tween 40	1-H5	D-psicose
1-A6	D-galactose	1-D11	Sucrose	1-H8	Pyruvic acid
1-A7	L-aspartic acid	1-D12	Uridine	1-H10	D-galacturonic acid
1-A8	L-proline	1-E1	L-glutamine	2-B1	N-acetyl-D-galactosamine
1-A9	D-alanine	1-E3	D-glucose-1-phosphate	2-B7	L-arabitol
1-A10	D-trehalose	1-E4	D-fructose-6-phosphate	2-B8	Arbutin
1-A11	D-mannose	1-E5	Tween 80	2-B10	I-erythritol
1-B01	D-serine	1-E8	β-methyl-D-glucoside	2-D2	Salicin
1-B2	D-sorbitol	1-E9	Adonitol	2-D8	Xylitol
1-B3	Glycerol	1-E10	Maltotriose	2-E8	β-hydroxybutyric acid
1-B4	L-fucose	1-E11	2-deoxyadenosine	2-E9	γ-hydroxybutyric acid
1-B5	D-glucuronic acid	1-E12	Adenosine	2-E10	α-ketovaleric acid
1-B6	D-gluconic acid	1-F2	Citric acid	2-F1	D-lactic acid methyl ester
1-B9	L-lactic acid	1-F3	Myo-inositol	2-F2	Malonic acid
1-B11	D-mannitol	1-F5	Fumaric acid	2-F10	Succinamic acid
1-B12	L-glutamic acid	1-F9	Glycolic acid	2-G3	N-acetyl-L-glutamic acid
1-C1	D-glucose-6-phosphate	1-F12	Inosine	2-G8	Hydroxy-L-proline
1-C3	D, L-malic acid	1-G3	L-serine	2-G9	L-isoleucine
1-C5	Tween 20	1-G5	L-alanine	2-G10	L-leucine
1-C6	L-rhamnose	1-G6	L-alanyl-glycine	2-G12	L-methionine
1-C7	D-fructose	1-G8	N-acetyl-β-D-mannosamine	2-H2	L-phenylalanine
1-C9	α-D-glucose	1-G11	D-malic acid	2-H3	L-pyroglutamic acid
1-C10	Maltose	1-G12	L-malic acid	2-H7	D, L-octopamine
1-C12	Thymidine	1-H2	p-hydroxyphenylacetic acid		

“1” and “2” indicate PM1 and PM2 metabolic plates, respectively.

#### Phenotyping of N source metabolism in Zr60

On the PM3 and PM6–8 N source assay plates, Zr60 metabolized 359 N sources (80 on PM3, 95 on PM6, 95 on PM7, and 89 on PM8). Of the 95 amino acid N sources on the PM3 plate, 15 were not metabolized, while 60 were efficiently utilized (Figure 11 PM3, Table 4). Among the 285 peptide N sources on the PM6–8 plates, only 6 were not metabolized (Figure 11 PM8, Table 4), whereas 267 were efficiently utilized (Figure 11 PM6–8).

**Table 4:** Substrates in PM3–PM8 microplates not metabolized by *A. faecalis* Zr60.

Well	Substrate	Well	Substrate	Well	Substrate
3-A4	Nitrate	3-D8	Ethylamine	3-F11	Uridine
3-A6	Biuret	3-D9	Ethanolamine	8-F7	D-Ala-D-Ala
3-C5	D-aspartic acid	3-E1	Histamine	8-F11	D-Leu-Gly
3-D2	N-phthaloyl-L-glutamic acid	3-E5	Formamide	8-G5	Gly-D-Ser
3-D5	Methylamine	3-E9	D-galactosamine	8-G6	Gly-D-Thr
3-D6	N-amylamine	3-F9	Thymidine	8-G7	Gly-D-Val
3-D7	N-butylamine	3-F10	Uracil	8-G12	D-Ala-Gly-Gly

“3” and “8” indicate PM3 and PM8 metabolic plates, respectively.

#### Metabolic phenotyping of P and S sources and associated biosynthetic pathways in Zr60

Among the 59 P sources tested (Figure 11 PM4 A02–E12 wells), Zr60 metabolized all, with 52 utilized efficiently. Similarly, among the 35 S sources tested (Figure 11 PM4 F02–H12 wells), Zr60 metabolized all, with 34 efficiently utilized. On the PM5 biosynthetic pathway assay plate (Figure 11 PM5), 94 biosynthetic pathways were tested, and Zr60 efficiently utilized all of them.

#### Phenotyping of osmolality and pH metabolism in Zr60

Zr60 survived in high concentrations of sodium chloride (5%), sodium sulfate (5%), sodium phosphate (pH 7, 200 mM), potassium chloride (6%), ethylene glycol (20%), urea (5%), ammonium sulfate (pH 8, 100 mM), sodium nitrate (100 mM), and sodium nitrite (100 mM). However, it did not survive in 6–10% sodium chloride, 3–6% sodium formate, 2–12% sodium lactate, or 50–200 mM sodium benzoate at pH 5.2 (Figure 11 PM9, Table 5). Zr60 grew within a pH range of 5–10, with optimal growth at pH 9. At pH 4.5, growth was supported by most amino acids tested except L-alanine, L-arginine, and L-asparagine. At pH 9.5, growth was normal with all amino acids tested except L-tryptophan and tyramine (Figure 11 PM10, Table 6). Additionally, wells B1–D12 and E1–G12 of the PM10 plate were used to assess amino acid decarboxylase and deaminase activities of Zr60 at pH 4.5 and 9.5, respectively. The strain exhibited 49% decarboxylase activity and 94% deaminase activity in the presence of most amino acids. Furthermore, Zr60 metabolized caprylate, *α*-D-glucoside, *β*-D-glucoside, α-D-galactoside, β-D-galactoside, α-D-glucuronide, β-D-glucuronide, β-D-glucosaminide, β-D-galactosaminide, α-D-mannoside, phosphate, and sulfate (Figure 11 PM10 H1–H12 wells, Table 6).

**Table 5:** Metabolic profiling of *A. faecalis* Zr60 on the Biolog PM9 plate.

Well	Substrate	Result	Well	Substrate	Result	Well	Substrate	Result	Well	Substrate	Result
9-A1	NaCl 1%	+	9-C1	NaCl 6% + KCl	+	9-E1	Sodium formate 1%	+	9-G1	Sodium phosphate pH 7, 20 mM	+
9-A2	NaCl 2%	+	9-C2	NaCl 6% + L-proline	+	9-E2	Sodium formate 2%	+	9-G2	Sodium phosphate pH 7, 50 mM	+
9-A3	NaCl 3%	+	9-C3	NaCl 6% + N-acetyl L-glutamine	+	9-E3	Sodium formate 3%	−	9-G3	Sodium phosphate pH 7, 100 mM	+
9-A4	NaCl 4%	+	9-C4	NaCl 6% + ß-glutamic acid	+	9-E4	Sodium formate 4%	−	9-G4	Sodium phosphate pH 7, 200 mM	+
9-A5	NaCl 5%	+	9-C5	NaCl 6% + γ-aminobutyric acid	+	9-E5	Sodium formate 5%	−	9-G5	Sodium benzoate pH 5.2, 20 mM	+
9-A6	NaCl 5.5%	+	9-C6	NaCl 6% + glutathione	+	9-E6	Sodium formate 6%	−	9-G6	Sodium benzoate pH 5.2, 50 mM	−
9-A7	NaCl 6%	−	9-C7	NaCl 6% + glycerol	+	9-E7	Urea 2%	+	9-G7	Sodium benzoate pH 5.2, 100 mM	−
9-A8	NaCl 6.5%	−	9-C8	NaCl 6% + trehalose	−	9-E8	Urea 3%	+	9-G8	Sodium benzoate pH 5.2, 200 mM	−
9-A9	NaCl 7%	−	9-C9	NaCl 6% + trimethylamine-N-oxide	+	9-E9	Urea 4%	+	9-G9	Ammonium sulfate pH 8, 10 mM	+
9-A10	NaCl 8%	−	9-C10	NaCl 6% + trimethylamine	−	9-E10	Urea 5%	+	9-G10	Ammonium sulfate pH 8, 20 mM	+
9-A11	NaCl 9%	−	9-C11	NaCl 6% + octopine	−	9-E11	Urea 6%	−	9-G11	Ammonium sulfate pH 8, 50 mM	+
9-A12	NaCl 10%	−	9-C12	NaCl 6% + trigonelline	−	9-E12	Urea 7%	−	9-G12	Ammonium sulfate pH 8, 100 mM	+
9-B1	NaCl 6%	+	9-D1	Potassium chloride 3%	+	9-F1	Sodium lactate 1%	+	9-H1	Sodium nitrate 10 mM	+
9-B2	NaCl 6% + betaine	+	9-D2	Potassium chloride 4%	+	9-F2	Sodium lactate 2%	−	9-H2	Sodium nitrate 20 mM	+
9-B3	NaCl 6% + N-N dimethyl glycine	+	9-D3	Potassium chloride 5%	+	9-F3	Sodium lactate 3%	−	9-H3	Sodium nitrate 40 mM	+
9-B4	NaCl 6% + sarcosine	+	9-D4	Potassium chloride 6%	+	9-F4	Sodium lactate 4%	−	9-H4	Sodium nitrate 60 mM	+
9-B5	NaCl 6% + dimethyl sulphonyl propionate	+	9-D5	Sodium sulfate 2%	+	9-F5	Sodium lactate 5%	−	9-H5	Sodium nitrate 80 mM	+
9-B6	NaCl 6% + MOPS	+	9-D6	Sodium sulfate 3%	+	9-F6	Sodium lactate 6%	−	9-H6	Sodium nitrate 100 mM	+
9-B7	NaCl 6% + ectoine	+	9-D7	Sodium sulfate 4%	+	9-F7	Sodium lactate 7%	−	9-H7	Sodium nitrite 10 mM	+
9-B8	NaCl 6% + choline	−	9-D8	Sodium sulfate 5%	+	9-F8	Sodium lactate 8%	−	9-H8	Sodium nitrite 20 mM	+
9-B9	NaCl 6% + phosphoryl choline	−	9-D9	Ethylene glycol 5%	+	9-F9	Sodium lactate 9%	−	9-H9	Sodium nitrite 40 mM	+
9-B10	NaCl 6% + creatine	−	9-D10	Ethylene glycol 10%	+	9-F10	Sodium lactate 10%	−	9-H10	Sodium nitrite 60 mM	+
9-B11	NaCl 6% + creatinine	+	9-D11	Ethylene glycol 15%	+	9-F11	Sodium lactate 11%	−	9-H11	Sodium nitrite 80 mM	+
9-B12	NaCl 6% + L-carnitine	−	9-D12	Ethylene glycol 20%	+	9-F12	Sodium lactate 12%	−	9-H12	Sodium nitrite 100 mM	+

“+”, metabolized; “–”, not metabolized.

**Table 6:** Metabolic profiling of *A. faecalis* Zr60 on the Biolog PM10 plate.

Well	Substrate	Result	Well	Substrate	Result	Well	Substrate	Result	Well	Substrate	Result
10-A1	pH 3.5	−	10-C1	pH 4.5 + L-methionine	−	10-E1	pH 9.5	+	10-G1	pH 9.5 + anthranilic acid	+
10-A2	pH 4	−	10-C2	pH 4.5 + L-phenylalanine	−	10-E2	pH 9.5 + L-phenylalanine	+	10-G2	pH 9.5 + L-norleucine	+
10-A3	pH 4.5	−	10-C3	pH 4.5 + L-proline	+	10-E3	pH 9.5 + L-arginine	+	10-G3	pH 9.5 + L-norvaline	+
10-A4	pH 5	+	10-C4	pH 4.5 + L-serine	+	10-E4	pH 9.5 + L-asparagine	+	10-G4	pH 9.5 + agmatine	+
10-A5	pH 5.5	+	10-C5	pH 4.5 + L-threonine	+	10-E5	pH 9.5 + L-aspartic acid	+	10-G5	pH 9.5 + cadaverine	+
10-A6	pH 6	+	10-C6	pH 4.5 + L-tryptophan	−	10-E6	pH 9.5 + L-glutamic acid	+	10-G6	pH 9.5 + putrescine	+
10-A7	pH 7	+	10-C7	pH 4.5 + L-tyrosine	+	10-E7	pH 9.5 + L-glutamine	+	10-G7	pH 9.5 + histamine	+
10-A8	pH 8	+	10-C8	pH 4.5 + L-valine	+	10-E8	pH 9.5 + glycine	+	10-G8	pH 9.5 + phenylethylamine	+
10-A9	pH 8.5	+	10-C9	pH 4.5 + hydroxy-L-proline	+	10-E9	pH 9.5 + L-histidine	+	10-G9	pH 9.5 + tyramine	−
10-A10	pH 9	+	10-C10	pH 4.5 + L-ornithine	−	10-E10	pH 9.5 + L-isoleucine	+	10-G10	pH 9.5 + creatine	+
10-A11	pH 9.5	+	10-C11	pH 4.5 + L-homoarginine	−	10-E11	pH 9.5 + L-leucine	+	10-G11	pH 9.5 + trimethyl amine-N-oxide	+
10-A12	pH 10	+	10-C12	pH 4.5 + L-homoserine	−	10-E12	pH 9.5 + L-lysine	+	10-G12	pH 9.5 + urea	+
10-B1	pH 4.5	−	10-D1	pH 4.5 + anthranilic acid	−	10-F1	pH 9.5 + L-methionine	+	10-H1	X-caprylate	+
10-B2	pH 4.5 + L-alanine	−	10-D2	pH 4.5 + L-norleucine	−	10-F2	pH 9.5 + L-phenylalanine	+	10-H2	X-α-D-glucoside	+
10-B3	pH 4.5 + L-arginine	−	10-D3	pH 4.5 + L-norvaline	+	10-F3	pH 9.5 + L-proline	+	10-H3	X-ß-D-glucoside	+
10-B4	pH 4.5 + L-asparagine	−	10-D4	pH 4.5 + α-amino-N-butyric acid	+	10-F4	pH 9.5 + L-serine	+	10-H4	X-α-D-galactoside	+
10-B5	pH 4.5 + L-aspartic acid	−	10-D5	pH 4.5 + p-aminobenzoate	−	10-F5	pH 9.5 + L-threonine	+	10-H5	X-ß-D-galactoside	+
10-B6	pH 4.5 + L-glutamic acid	−	10-D6	pH 4.5 + L-cysteic acid	+	10-F6	pH 9.5 + L-tryptophan	−	10-H6	X-α-D-glucuronide	+
10-B7	pH 4.5 + L-glutamine	+	10-D7	pH 4.5 + D-lysine	+	10-F7	pH 9.5 + L-tyrosine	+	10-H7	X-ß-D-glucuronide	+
10-B8	pH 4.5 + glycine	+	10-D8	pH 4.5 + 5-hydroxy lysine	+	10-F8	pH 9.5 + L-valine	+	10-H8	X-ß-D-glucosaminide	+
10-B9	pH 4.5 + L-histidine	−	10-D9	pH 4.5 + 5-hydroxy tryptophan	+	10-F9	pH 9.5 + hydroxy-L-proline	+	10-H9	X-ß-D-galactosaminide	+
10-B10	pH 4.5 + L-isoleucine	−	10-D10	pH 4.5 + D, L-diaminopimelic acid	+	10-F10	pH 9.5 + L-ornithine	+	10-H10	X-α-D-mannoside	+
10-B11	pH 4.5 + L-leucine	−	10-D11	pH 4.5 + trimethyl amine-N-oxide	−	10-F11	pH 9.5 + L-homoarginine	+	10-H11	X-PO_4_	+
10-B12	pH 4.5 + L-lysine	+	10-D12	pH 4.5 + urea	+	10-F12	pH 9.5 + L-homoserine	+	10-H12	X-SO_4_	+

“+”, metabolized; “–”, not metabolized.

## Discussion

Tobacco brown spot is one of the most threatening diseases in tobacco cultivation. Screening antagonistic bacteria has become an essential component of biological control for this disease and a major research focus in recent years (Zhang and Guo, 2019). Numerous studies have reported the use of antagonistic bacteria to combat brown spot. For instance, Luo et al. (2014) used *A. alternata* as a screening indicator, applying plate confrontation for primary screening and fermentation supernatant for secondary screening. They isolated three *Bacillus* strains antagonistic to tobacco brown spot, with strain K11 achieving a mycelial radial growth inhibition rate of 63.64% and its fermentation broth supernatant showing an inhibition rate of 67.44%. Similarly, Ma et al. (2012) isolated 10 *Bacillus* strains with antagonistic activity from tobacco rhizosphere soil and leaves. Among them, strain M-07 exhibited the strongest effect, forming an inhibition zone 14.2 mm wide. Other reported biocontrol bacteria effective against tobacco brown spot include *Bacillus velezensis*, *Pseudomonas* spp., and *Streptomyces* spp. However, reports on *A. faecalis* remain limited.

In the growth-promoting activity tests of this study, *A. faecalis* exhibited the ability to solubilize inorganic P and produce siderophores. Similarly, Liu et al. (2012) reported that *Alcaligenes* spp. possess phosphate-solubilizing capabilities. Zhang et al. (2008) demonstrated that certain *Alcaligenes* spp. produce siderophores that enhance plant growth under stressful conditions. Kang et al. (2015) highlighted the ability of *A. faecalis* to produce IAA. Joseph et al. (2007) investigated the growth-promoting effects of *A. faecalis* on chickpeas, including IAA production and phosphate solubilization. Rana and Shivay (2012) found that *A. faecalis* enhances nutrient absorption and improves yield in wheat. Collectively, these studies indicate that *A. faecalis* can effectively promote plant growth.

Herein, *A. faecalis* Zr60, a bacterium antagonistic to tobacco brown spot disease, was isolated from tobacco rhizospheric soil. This strain exhibited broad-spectrum antimicrobial activity and strong efficacy against tobacco brown spot. The metabolic phenotypic data obtained provide important theoretical insights for the future development and utilization of this biocontrol bacterium. Our results showed high inhibition rates of Zr60 against *A. alternata* on plates and in fermentation broth. However, its field control efficacy requires further investigation. Similar to other biocontrol bacteria, the effectiveness of Zr60 in the field is influenced by factors such as temperature, climate, and soil composition, which affect its survival, colonization ability, and production of antibacterial compounds under natural conditions (Weller, 1988; Haas and Keel, 2003). Metabolic phenotyping analysis identified the preferred C, N, P, and S sources for Zr60 as well as substrates unfavorable for its metabolism. These findings provide valuable insights into the optimal cultivation conditions for Zr60 and help clarify the factors that either induce or inhibit cell differentiation, thereby maximizing its biocontrol efficacy. Such optimization could broaden its applicability to other plant diseases and support improvements in its degradation capacity, facilitating industrial production. The 952 metabolic phenotypic data points of Zr60 in this study were generated using the Biolog phenotype testing platform. However, questions remain regarding the consistency of actual metabolic activity and substrate utilization compared with Biolog PM results. Additionally, the relationship between substrate concentrations and metabolic capacity as well as the mechanisms by which these substrates influence the metabolism of Zr60 require further investigation. More in-depth research is needed to evaluate substrate utilization for developing biopesticides or biofertilizers. Moreover, the biological control of tobacco brown spot involves complex interactions among the tobacco plant, pathogen, *A. faecalis*, and multiple environmental factors. Therefore, understanding only the metabolic phenotype of Zr60 is insufficient. It is also essential to characterize the metabolic phenotype of the tobacco brown spot pathogen and compare the metabolic profiles of the two microorganisms. Furthermore, tobacco root exudates and soil contain diverse metabolic substrates, including different C, N, P, and S sources. Understanding the abundance of these substrates and their effects on Zr60 growth can further enhance its functional performance in biocontrol applications.

## Conclusion

Herein, strain Zr60, with a robust inhibitory effect against tobacco brown spot disease, was isolated from the rhizospheric soil of healthy tobacco plants. It was identified as *A. faecalis* through morphological observation and 16S rRNA sequence analysis. The strain exhibited inhibition rates of 81.93% on plates and 87.47% in fermentation broth. Moreover, it demonstrated broad-spectrum *in vitro* antagonistic activity, with an average inhibition rate of 75.64% against eight phytopathogens, indicating its potential to effectively combat common plant pathogens. Analyses of the growth-promoting activity and biological characteristics of Zr60 revealed its ability to solubilize inorganic P and produce siderophores. The optimal growth temperature was 28 °C, and the optimal medium was LB broth. Phenomics analysis generated 758 metabolic phenotypic data points for Zr60, including 190\u00B0C source, 380 N source, 59 P source, 35 S source, and 94 biosynthetic pathway phenotypes. This strain was capable of metabolizing 95% of C, 94% of N, 100% of P, and 100% of S sources as well as activating all tested biosynthetic pathways. Osmotic pressure and pH metabolic phenotype analysis showed that Zr60 adapted to 65% of osmotic environments, with an optimal pH of 9. Zr60 also exhibited 49% amino acid decarboxylase activity and 94% amino acid deaminase activity, further highlighting its metabolic versatility.

## Data Availability

All data generated or analyzed during this study are included in this article. The 16S rRNA sequence of *A. faecalis* Zr60 has been deposited in the NCBI database under GenBank accession number PP535405.1.

## Footnotes

1
www.ncbi.nlm.nih.gov

2
https://www.megasoftware.net/
